# Expression of Wnt and TGF-Beta Pathway Components during Whole-Body Regeneration from Cell Aggregates in Demosponge *Halisarca dujardinii*

**DOI:** 10.3390/genes12060944

**Published:** 2021-06-20

**Authors:** Ilya Borisenko, Fyodor V. Bolshakov, Alexander Ereskovsky, Andrey I. Lavrov

**Affiliations:** 1 Faculty of Biology, Department of Embryology, Saint-Petersburg State University, 199034 Saint-Petersburg, Russia; alexander.ereskovsky@imbe.fr; 2 Biological Faculty, Pertsov White Sea Biological Station, Lomonosov Moscow State University, 119234 Moscow, Russia; fedbolsh@mail.ru (F.V.B.); lavrovai.bio@yandex.ru (A.I.L.); 3 Institut Méditerranéen de Biodiversité et d’Ecologie Marine et Continentale (IMBE), Aix Marseille University, CNRS, IRD, Station Marine d’Endoume, Rue de la Batterie des Lions, Avignon University, 13007 Marseille, France; 4 Evolution of Morphogenesis Laboratory, Koltzov Institute of Developmental Biology of Russian Academy of Sciences, 119334 Moscow, Russia

**Keywords:** primmorph, axial patterning, signaling pathways, Wnt, TGF-beta, whole-body regeneration

## Abstract

The phenomenon of whole-body regeneration means rebuilding of the whole body of an animal from a small fragment or even a group of cells. In this process, the old axial relationships are often lost, and new ones are established. An amazing model for studying this process is sponges, some of which are able to regenerate into a definitive organism after dissociation into cells. We hypothesized that during the development of cell aggregates, primmorphs, new axes are established due to the activation of the Wnt and TGF-beta signaling pathways. Using in silico analysis, RNA-seq, and whole-mount in situ hybridization, we identified the participants in these signaling pathways and determined the spatiotemporal changes in their expression in demosponge *Halisarca dujardinii*. It was shown that Wnt and TGF-beta ligands are differentially expressed during primmorph development, and transcripts of several genes are localized at the poles of primmorphs, in the form of a gradient. We suppose that the Wnt and TGF-beta signaling cascades are involved in the initial axial patterning of the sponge body, which develops from cells after dissociation.

## 1. Introduction

Signaling pathways Wnt and TGF-beta are involved in the formation of axes in multicellular animals: they provide axial patterning in developing embryos and support it in adult animals. In addition to development, both signaling cascades are involved in the regeneration of multicellular animals of different levels of complexity—from cnidarians to vertebrates. Wnt and TGF-beta pathways regulate cell proliferation, migration, differentiation, and apoptosis during various regenerative processes [1,2,3,4,5].

Sponges (Porifera) occupy a key position at the base of the phylogenetic tree of animals. With this in mind, they are essential for understanding the origin and evolution of multicellular body patterning. The axes in the body of sponges are clearly different from those in other multicellular animals because most sponges have a polarity axis but lack an axis of symmetry [6]. Interestingly, sponges have genes of the Wnt and TGF-beta pathways, and these genes are even involved in establishing axes in embryo and adult bodies. Ligands of both pathways have dynamic expression patterns during embryonic development and in the larva of *Amphimedon queenslandica* and *Sycon ciliatum*. In addition, in the adult *S. ciliatum*, the expression of several Wnt and TGF-beta is confined to the osculum and the entrances of the radial choanocyte chambers, i.e., axial structures [7,8,9].

However, the possible involvement of Wnt and TGF-beta in regeneration after injury in sponges has not been studied. Experiments with reparative regeneration are convenient only for sponges with a clear morphological polarity because, in this case, we can assume what place an excised area occupies with respect to the axes of the body. In massive multioscular leuconoid sponges, it is difficult to understand what position on the axis an excised body fragment occupied. That complicates the interpretation of the experiment results. In this case, another type of regeneration, whole-body regeneration (WBR) after tissue dissociation, i.e., cell reaggregation, provides a convenient model for studying polarity formation.

The original anatomical structure and patterning of the sponge body are completely disrupted during tissue dissociation. During the process of cell reaggregation, separated cells coalesce, forming structureless primary multicellular aggregates. Subsequently, these aggregates undergo a transition to radially symmetric, developing primmorphs with an aquiferous system, and then to a reconstructed functional sponge with a polarity axis formed de novo. This model has been thoroughly described at the morphological level in some sponge species (reviewed in [10,11]) but studied from the molecular point of view only in one species of calcareous sponges [12].

We began to study the participation of Wnt and TGF-beta signaling pathways in the cell reaggregation in demosponge *Halisarca dujardinii* with identification of pathways’ components in the sponge transcriptome, subsequent domain and phylogenetic analysis of orthologous proteins, and evaluation of gene expression levels at different stages of reaggregation using RNA-seq and whole-mount in situ hybridization (WMISH). Our preliminary results, including the first WMISH data on primmorphs, show differential dynamic expression of Wnt and TGF-beta components at different stages of reaggregation, as well as a polar localization for several transcripts.

## 2. Materials and Methods

### 2.1. Animals and Microscopy

Sponges *Halisarca dujardinii* Johnston, 1842, were collected in Kandalaksha Bay of the White Sea in the environs of the Pertsov White Sea Biological Station (66°34′ N, 33°08′ E) and in the Chupa Inlet near the Sredniy Island (66°15′ N, 33°05′ E) from the upper subtidal zone at 0–5 m depth. Before the experiments, all sponges were maintained in a laboratory aquarium with natural seawater and biological filters at 6–10 °C no longer than 24 h.

Sponge tissues were dissociated by mechanical squeezing through 50-µm nylon mesh into vessels with filtered sterile seawater (FSW). The FSW was used in the dissociation procedure and during subsequent cell cultivation to avoid additional contamination. Water was sterilized with syringe filter units 0.22 µm (Sartorius). Obtained cell suspensions were diluted with FSW up to concentrations 1–3 × 10^7^ cells/mL and maintained in FSW under 8–12 °C. Half of the culture medium was replaced with fresh FSW every 2 days [10]. Each cell culture was checked and photographed daily over the whole period of cultivation using stereomicroscope Leica M165FC (Leica) equipped with digital camera Leica DFC 420 and application Leica LAS Store and Recall v.4.2. For histological studies, aggregates were fixed with 2.5% glutaraldehyde and 1% OsO_4_ in modified 0.1 M Na-Cacodylate buffer (0.1 M Na-Cacodylate, 85.55 mM NaCl, 5 mM CaCl2, 5 mM MgCl2; pH 7.0–7.5) and embedded in Epon/Araldite epoxy embedding media as described previously [10]. According to in vivo morphology and histological structure of aggregates, six stages of cell reaggregation were distinguished: primary multicellular aggregates (PMAs), early-stage primmorphs (ESPs), true primmorphs (TPs), developing primmorphs (DPs), progressed developing primmorphs (PDP), and reconstructed functional sponges (FSs).

### 2.2. RNA-Seq and In Silico Analysis

We used the *H. dujardinii* transcriptome, which was previously sequenced using Illumina HiSeq 2500 and assembled onto contigs [13]. The RNA for reference transcriptome assembly was extracted from adult sponges and larvae.

Search for signaling pathway components was performed using a reciprocal BLAST approach. Sponge, cnidarian, and bilaterian gene orthologs were used in tblastn search of the *H. dujardinii* transcriptome assembly. Putative positive matches were then searched for conserved domains by HMMER 3.3.2 (hmmscan) against Pfam database and aligned to orthologs from other organisms. Alignments were performed using MUSCLE and then manually corrected (Supplementary Files S1–S5). For Bayesian inference, we used MrBayes 3.2.7 [14], using the mixed model with four independent runs of two million generations. For maximum likelihood analysis, we used RAxML 8.0 [15] with model selection by ProtTest3 [16]. Consensus trees were visualized in FigTree 1.4.4. List of accession numbers for sources is provided in Supplementary File S6. Identified sequences of *H. dujardinii* were submitted to GenBank with accession numbers MZ042492-MZ042530.

For RNA-seq analysis, RNA was extracted from aggregates at stages of ESP/TP, 1 day post dissociation (dpd), and DP, 6 dpd. Aggregates were rinsed several times with FSW and processed with RNeasy kit (QIAGEN). cDNA libraries were constructed and sequenced using Illumina HiSeq 4000 at Genomics Core of Research Technology Support Facility (Michigan State University). Paired 150 bp reads yield was 109 mln for 1-dpd samples and 108 mln for 6-dpd samples. Reads were pseudoaligned with Kallisto 0.42.4 [17] to a reference transcriptome, and differential expression analysis was performed in edgeR 3.12 in R [18].

### 2.3. Whole-Mount In Situ Hybridization (WMISH)

For WMISH, aggregates were fixed in MOPS and processed as described elsewhere [19] with minor modifications. Aggregates at approximately one developmental stage were manually collected from a culture. Fixed aggregates were stored at −20 °C in 70% EtOH. Due to fragility of material, rehydration from 70% EtOH to PTw buffer was performed drop-by-drop during 30–90 min. Tissue was digested with proteinase K for 10 min at 37 °C and concentration 5 µg/mL. Fragments of *TGF-beta* were amplified from cDNA and cloned in pAL-2T vector (Evrogen). Inserts were verified by Sanger sequencing and used for probe synthesis. An antisense digoxigenin-labeled RNA probe was made using in vitro transcription with DIG RNA labeling mix (Roche) and appropriate RNA-polymerase (Thermo Scientific). Some specimens after WMISH were dehydrated in ethanol and embedded in glycol methacrylate according to a published protocol [20]. Sections with thickness 5–7 µm were cut by glass knife in Leica UC7 ultramicrotome.

## 3. Results

### 3.1. Wnt Pathway Components in Halisarca dujardinii

We have previously identified Wnt ligands in the *H. dujardinii* transcriptome and described their phylogenetic position as well as expression in the adult sponge and larva [13]. We found 10 Wnt ligands containing conserved cysteine residues and an RWNC motif. Phylogenetic analysis showed that only one of them could be close to the Wnt (namely AquWntC) of another demosponge, *Amphimedon queenslandica*, and thus the molecules were named alphabetically, starting with HduWntC. Here, we searched the transcriptome for the main participants in the intracellular signal transduction in the Wnt cascade: Frizzled receptors and LRP coreceptors, a secondary messenger Dishevelled, transcriptional cofactors (beta-catenin, TCF, Groucho), a protein complex responsible for beta-catenin degradation, and enzymes responsible for Wnt ligand maturation (porcupine and Wntless).

**Figure 1:**
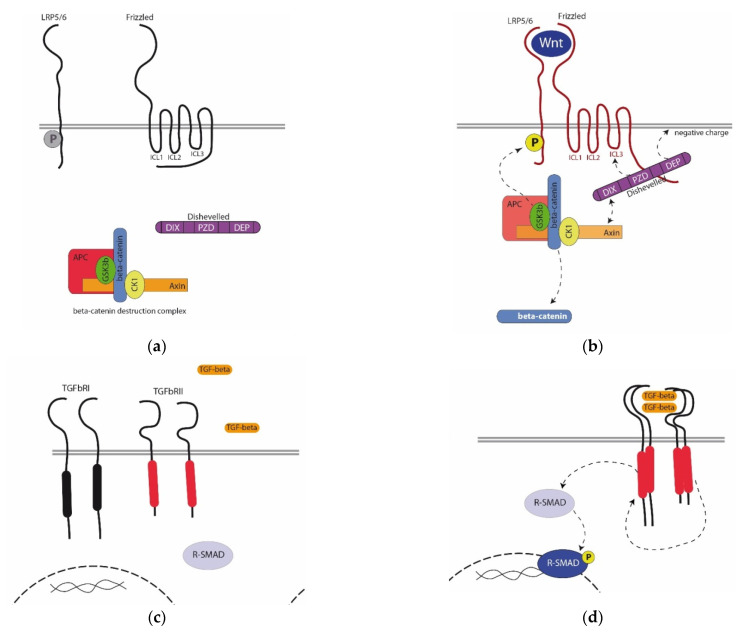
(**a**,**b**) β-catenin-dependent Wnt cascade. In the absence of a signal (ligand) in the intercellular space (the OFF state, (**a**), the cytoplasmic regions of the Frizzled are masked, and the sites at the C-end of the LRP5/6 are not phosphorylated (inactive receptors are shown in black). When the ligand binds to the receptors (ON state, activated receptors are shown in red; (**b**), Dishevelled binding sites are opened at the C-terminus of Frizzled and ICL3, due to which Dishevelled is attracted to the receptor. Dashed arrows show the interaction of Dishevelled domains with different parts of Frizzled. Due to the recruitment of bCDC to the receptor, GSK3b phosphorylates threonine and serine amino acid residues in the cytoplasmic part of LRP5/6 (dashed arrow). As a result, the bCDC molders and the beta-catenin is released into the cytoplasm. (**c**,**d**) TGF-beta signaling pathway. In the absence of mature TGF-beta dimers, constitutively active Type II receptors (active kinase domains are shown in red) are separated from the Type I receptors (**c**). When the TGF dimer binds, the Type I and Type II receptors are assembled into a complex, and a chain of sequential phosphorylation leads to the phosphorylation of R-SMAD. It forms a dimer with Co-SMAD and is translocated into a nucleus.

Many models for the regulation of the activity of the Wnt cascade have been described. The simplest ON/OFF model is as follows. In the absence of a signal (OFF state), the pool of the beta-catenin is constantly degraded due to the beta-catenin destruction complex (bCDC) in the cytoplasm of a competent (responding to a signal) cell (Figure 1a). bCDC, consisting of the core proteins Axin and APC, glycogen synthase kinase-3 beta (GSK3b), and casein kinase 1 (CK1), phosphorylates cytoplasmic beta-catenin, which leads to its ubiquitin-dependent degradation in proteasomes. In the instructing (generating a signal) cell, prior to secretion, the Wnt ligand is transported and acylated in the Golgi apparatus by Wntless protein and palmitoleoyltransferase porcupine, respectively. Acylation is absolutely necessary for the binding of the Wnt ligand to the receptor. Blocking these enzymes by knockout or drugs makes signal transmission impossible. On the surface of a competent cell, the ligand binds to the Frizzled receptor and LRP5/6 coreceptor (Figure 1b). Frizzled is a seven-pass transmembrane protein, and LRP is a single-pass one. The cytoplasmic regions of both proteins contain sequences responsible for interaction with cytoplasmic signaling participants: the third intracytoplasmic loop (ICL3) and C-end of Frizzled responsible for interaction with Dishevelled protein, whereas the cytoplasmic end of LRP5/6 contains sites phosphorylated by GSK3b from bCDC. The binding of the ligand to the receptor and coreceptor leads to conformational changes in the cytoplasmic domains of the Frizzled and recruitment of Dishevelled from the cytoplasm. Dishevelled, in turn, recruits bCDC, resulting in its break into individual proteins and loss of the ability to phosphorylate beta-catenin. beta-catenin is preserved from degradation and migrates into a nucleus, regulating the expression of target genes as a transcriptional cofactor [21].

Using reciprocal BLAST, we identified six Frizzled paralogs, four SFRP paralogs, and one protein for each of LRP5/6, porcupine, Wntless, Dishevelled, APC, TCF, Groucho, GSK3b, CK1, and beta-catenin. No Axin, one of the core components of the bCDC, acting as a kernel in the assembly of the complex, was found in the *H. dujardinii* transcriptome.

**Figure 2:**
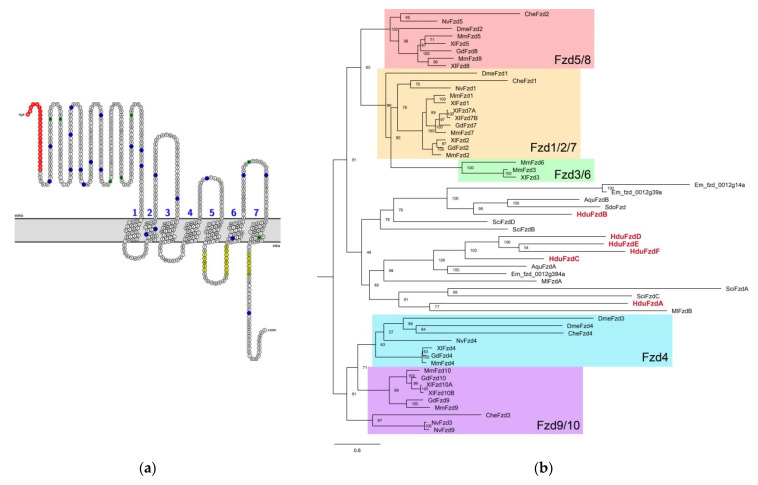

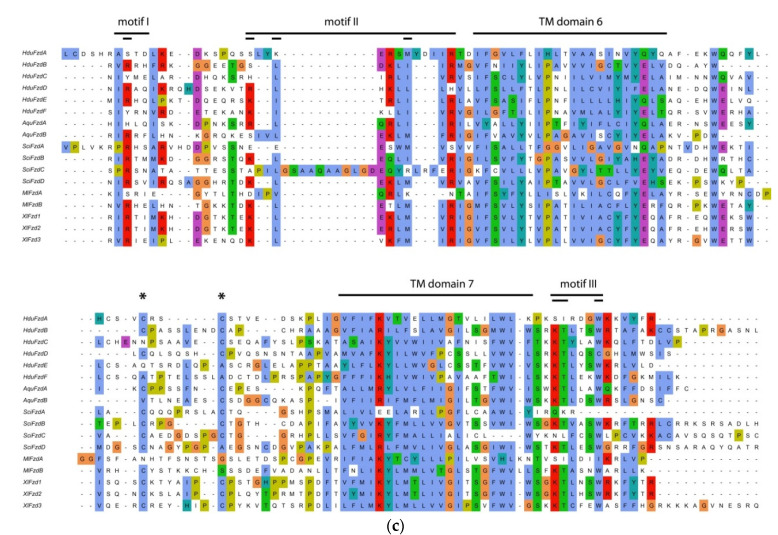
Frizzled receptors in *Halisarca dujardinii*. (**a**) HduFzdB topology. Extracellular CRD with blue cysteine residues is shown. Three conserved motifs responsible for interaction with Dishevelled are highlighted in yellow—motif I and II between 5th and 6th transmembrane domain, and motif III at C-end of protein. The signal peptide is red. (**b**) Unrooted ML tree of Frizzled from sponges, cnidarians, ctenophore, and bilaterians. *H. dujardinii* sequences are in red. (**c**) Alignment of Frizzled sequences from *H. dujardinii*, *Sycon ciliatum*, *Mnemiopsis leidyi,* and *Xenopus laevis* at the region of motifs I-III (in yellow in Figure 2a. Motifs are marked by a black line, the invariant amino acid residues—by a double line. Conserved cysteines in the extracellular loop between 6 and 7 TM domains are indicated by asterisks (*). Species designations: Aqu, *Amphimedon queenslandica*; Bfl, *Branchiostoma floridae*; Cel, *Caenorhabditis elegans*; Che, *Clytia hemisphaerica*; Cin, *Ciona intestinalis*; Dme, *Drosophila melanogaster*; Dre, *Danio rerio*; Em, *Ephydatia muelleri*; Gd, *Gallus domesticus*; Hro, *Halocynthia roretzi*; Hdu, *Halisarca dujardinii*; Hs, *Homo sapiens*; Ml, *Mnemiopsis leidyi*; Mm, *Mus musculus*; Nv, *Nematostella vectensis*; Pca, *Podocoryne cornea*; Rn, *Rattus norvegicus*; Sci, *Sycon ciliatum*; Sdo, *Suberites domuncula*; Sko, *Saccoglossus kowalevskii*; Spu, *Strongylocentrotus purpuratus*; Tad, *Trichoplax adhaerens*; Tca, *Tribolium castaneum*; Xl, *Xenopus laevis*.

The N-end of the Frizzled receptor facing the intercellular space contains an extended cysteine-rich domain (CRD) responsible for the binding of the Wnt ligand (Figure 2a).

All six *H. dujardinii* Frizzled have a secondary structure typical for this receptor: an extracellular CRD domain, seven transmembrane domains, conservatively located cysteines in loops between transmembrane domains, and motifs in intracellular regions responsible for interaction with Dishevelled (Figure 2a).

Phylogenetic analysis of the evolutionary relationships of the identified sponge *Frizzled* genes showed a situation similar to the position of Wnt ligands: we cannot assign them to any of the receptor families described in higher metazoans (Figure 2b). In this regard, by analogy with ligands, we named receptors in alphabetical order. 

In addition to available *Sycon ciliatum*, *A. queenslandica,* and *Suberites domuncula* sequences from sponges, and *Mnemiopsis leidyi* from Ctenophora, we identified *Frizzled* sequences in the recently sequenced demosponge *Ephydatia muelleri* genome [22]. Three sequences were obtained by tblastn with consequent HMMSCAN against Pfam database and TMHMM analysis. These three Frizzleds represent seven-pass transmembrane receptors with a CRD domain in the extracellular part of the peptide. 

The branching order of the different families is conserved between the different trees, and family groupings are generally well supported. There appear to be five bilaterian families: Fzd1/2/7, Fzd3/6, Fzd5/8, Fzd4, and Fzd9/10. The nomenclature of some analyzed nonbilaterian genes is misleading: *CheFzd2* and *DmeFzd2* fall into the Fzd5/8 branch, *DmeFzd3* fall into the Fzd4 family, and *CheFzd3* and *NveFzd3* fall into the Fzd9/10 family. The last two look like mistakes of the phylogenetical classification of *Clytia hemisphaerica*, *Nematostella vectensis*, and *Drosophila melanogaster* sequences because the Fzd3/6 family seems to be a chordate innovation [8].

Frizzled contains three motifs for interaction with the PZD domain of the Dishevelled protein. ICL3 of Frizzled contains two of these motifs—motif 1 (IRxV) and motif 2 (KLEKLMVR, Figure 2a,c). Most of the invariant amino acids in these motifs occupy positions in the alignments that are conservative with respect to vertebrates. There are two conserved cysteine residues between TM domains 6 and 7; the first of them is replaced with non-similar amino acid residue only in *HduFzdC* and *HduFzdE* (Figure 2c). The same substitution is observed in *FzdA* of the ctenophore *M. leidyi* [23]. The third motif (KTxxxW) is located in the free cytoplasmic C-end of Frizzled. Here invariant amino acid residues are substituted in *HduFzdA*, as well as in *MleFzdA* and *SciFzdA*. This Frizzled-specific motif has been shown to directly interact with the PDZ domain of Dishevelled [24]. Thus, in *H. dujardinii*, the majority of Frizzled proteins demonstrate conservatism at the level of functionally essential protein sequences ensuring signal transduction into the cytoplasm. *HduFzdA* seems to be an exception, as it contains several non-analogous substitutions in the motifs.

There are also secreted Frizzled-related proteins (SFRPs) that have a CRD but lack transmembrane domains. They represent soluble forms of the receptor secreted by a cell and act as ligand traps because they bind diffusing Wnt and prevent it from binding to functional receptors. We identified four such proteins that have a CRD with a high level of similarity to Frizzled. However, the number of CRD-containing proteins is large, and their targets are heterogeneous because interaction occurs between two cysteine-rich regions of the polypeptide chain. Probably other CRD-containing proteins can act as functional analogs of SFRPs, among the described ones (for example, Dickkopf) [25]. The regulation of signaling activity at the level of ligand–receptor binding is a rather broad topic, and thus we leave the analysis of SFRPs for further research.

**Figure 3:**
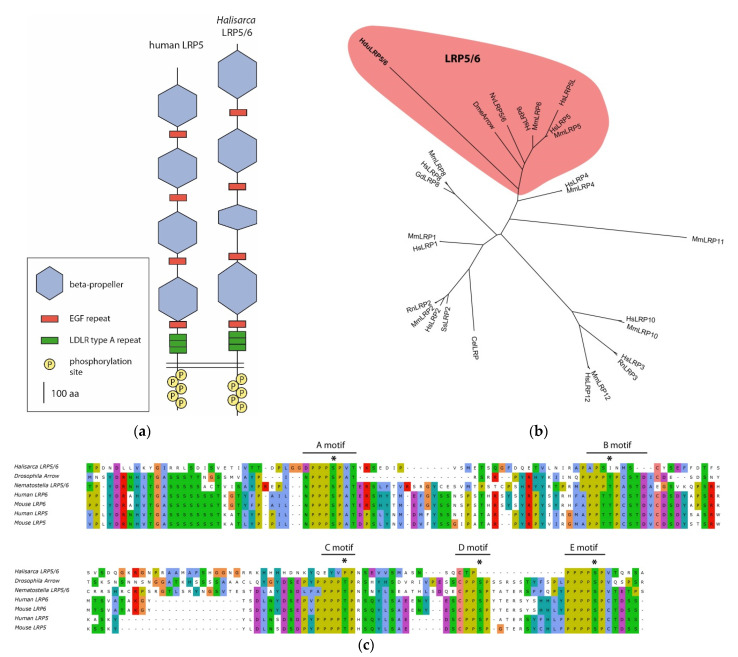
LRP5/6 protein in *Halisarca dujardinii*. (**a**) Comparison of domain organization in the human LRP5 and *H. dujardinii* LRP5/6 ortholog demonstrates conservative domain set and arrangement. (**b**) Unrooted ML tree indicates the phylogenetic position of *HduLRP5/6* close to bilaterian orthologs. (**c**) Alignment of the C-end of HduLRP5/6 with *Drosophila melanogaster*, *Nematostella vectensis*, and mammalian orthologs demonstrates five conserved motifs targeted for phosphorylation. Sites recognizable by GSK3b are indicated by asterisks (*).

The Wnt coreceptor belongs to the family of low-density lipoprotein receptors (LDLRs), only the 5 and 6 classes (LRP5/6) of which are involved in the Wnt cascade. LRP5/6 are characterized by a conservative set and arrangement of domains. We found several LDLRs and focused on them because LRP5/6 is responsible for recruiting cytoplasmic protein Axin—a core component of the bCDC. Due to the absence of Axin in *H. dujardinii*, it seemed important to understand whether it has a structurally and functionally conservative LRP5/6. Only one of the found LDLRs possessed the complete set of distinctive features of LRP5/6 (Figure 3a): the extracellular part of the receptor contains four tandems of a beta-propeller and EGF repeats. A beta-propeller is a domain that has a tertiary structure resembling a six-bladed propeller, with each blade consisting of four parallel beta-sheets. Each beta-propeller consists of five LDLR class B1 repeats. We were not able to identify all character repeats of class B1 with HMMSCAN, and therefore additionally we predicted the secondary structure of peptide with JPred4 [26]. The position and number of beta-sheet sets coincided with the expected position of beta-propellers in the peptide, and their predicted tertiary organization corresponds to a beta-propeller (Supplementary File S7). In the juxtamembrane region of the receptor, there are conserved three repeats of LDLR class A. The receptor’s cytoplasmic domain contains five PPPSPxS motifs, which are phosphorylated by GSK3b and CK1 upon activation of the cascade. These motifs mediate interaction with Axin [27,28,29,30,31]. Alignment with proteins of mammals, *D. melanogaster*, and *N. vectensis* shows that conservative phosphorylated amino acid residues are present in four out of five motifs (Figure 3c). In the phylodendrogram, the LRP5/6 of the *H. dujardinii* falls in the clade with the Arrow protein of *D. melanogaster*, LRP5/6 of the *N. vectensis*, and LRP classes 5 and 6 of mammals.

### 3.2. TGF-Beta Pathway Components in Halisarca dujardinii

The TGF-beta superfamily includes more than 30 structurally related proteins that are morphogens similarly to Wnt. Several families are distinguished in this superfamily: TGF-beta itself, several BMP families, Lefty, Activin/Inhibin, Vg1, a number of Gdf, and others. All ligands from TGF-beta superfamily synthesized as a precursor protein. This precursor contains three domains—the N-terminal signal peptide, propeptide (or latency-associated peptide), and the C-terminal mature peptide. During processing, the mature peptide is cleaved by Furin, a convertase, at the RxxR conserved site [32]. The mature peptide forms a dimer, which binds to the specific TGF-beta receptor (Figure 1c,d; [33]). The TGF-beta receptor is a single-pass membrane protein containing a cytoplasmic kinase domain with both serine/threonine kinase activity and tyrosine kinase activity [34]. There are two types of the TGF-beta receptors—Type II receptor, constitutively active, and Type I receptor. When the dimeric ligand binds, a complex is formed of two Type I receptor molecules and two Type II receptor molecules. Constitutively active Type II receptor then phosphorylates a TGF-b Type I receptor. Phosphorylated Type I receptors phosphorylate and thereby activate receptor-associated SMAD proteins (R-SMADs), including SMAD1/5 and SMAD2/3 [35,36]. R-SMAD proteins are composed of two main functional domains: the Mad-homology domains 1 and 2 (MH1 and MH2). Activated R-SMADs interact with the common-mediator SMAD (Co-SMAD, SMAD4) and become translocated to the nucleus. This SMAD protein complex then regulates the expression of TGF-beta target genes by interacting with transcription factors. Inhibitory SMADs (I-SMADs, SMAD-6) are able to compete with Co-SMADs in interaction with R-SMADs, thus inhibiting the signaling pathway.

**Figure 4:**
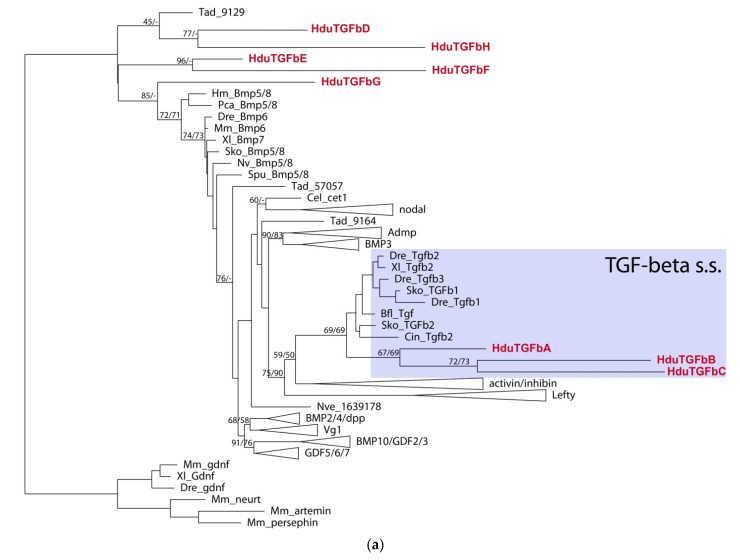

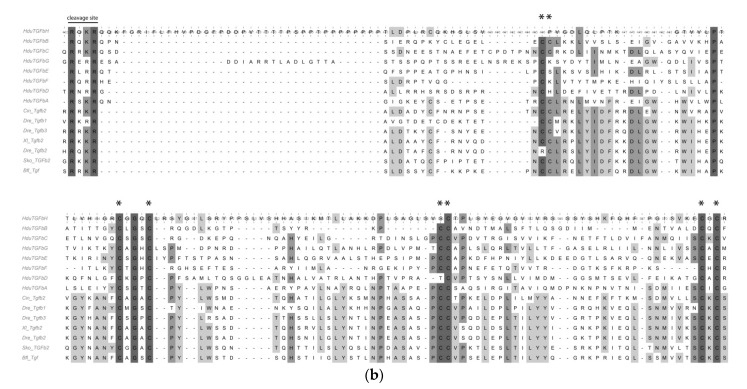
(**a**) TGF-beta ligand tree rooted with GDNF/artemin/persephin proteins. Some bilaterian families are collapsed (Lefty, Vg1, Nodal, BMPs). Only mature TGF-beta peptides were used in the analysis. Bootstrap support and Bayesian posterior probability are indicated as numerator and denominator at the nodes, respectively. Dash means that the node is not supported by analysis. *Halisarca dujardinii* sequences are highlighted in red. (**b**) Alignment of mature TGF-beta peptides from *H. dujardinii* and bilaterian TGF-beta s.s. family members. The cleavage site for Furin is shown, and eight conserved cysteine residues are indicated by asterisks (*).

We were able to identify and isolate eight putative TGF-beta ligands, six receptors, and six SMADs. Due to the relatively high divergence of the sponge sequences, only three ligands, *HduTGFbA*–*HduTGFbC*, could be ascribed to TGF-beta sensu stricto, while the rest five sequences (*HduTGFbD*–*HduTGFbH*) group as sister to the other TGF-beta families, with the closest TGF member being one of *Trichoplax adhaerens* ligands (Figure 4a). 

*HduTGFbA*, *HduTGFbB*, and *HduTGFbC* have eight cysteine residues, conserved in gene families of the TGF-beta-related clade (Figure 4b). *HduTGFbG*, *HduTGFbE*, and *HduTGFbF* have seven conserved cysteine residues, while *HduTGFbD* has only six, and *HduTGFbH* missed first, second, and fifth cysteine residues and have only five of them. Interestingly, when we ran an analysis on a full TGF-beta precursor with both the propeptide and the peptide domain, only *HduTGFbA* fell into the TGF-beta s.s. clade and the rest of the ligand groups at the base of the TGF-beta tree. 

Three pairs of genes appear to be relatively recent tandem duplications since they group closely together and are located adjacent to each other on the same contig of the genome draft: *HduTGFbB*/*HduTGFbC*, *HduTGFbD*/*HduTGFbH*, and *HduTGFbE*/*HduTGFbF*. The rest two ligands, *HduTGFbA* and *HduTGFbG*, are located on separate contigs (data not shown).

Sequence analyses using SignalP and HMMSCAN predicted signal peptides and TGF-beta peptides for *HduTGFbA*–*HduTGFbD* and *HduTGFbG*–*HduTGFbH*. We suggest that the propeptides are missing in these cases or are highly divergent and not detected by homology searches. For *HduTGFbE* and *HduTGFbF*, a propeptide and TGF-b peptide are predicted, but the signal sequence is not. The cleavage site for furin protease (RxxR) at the border of the mature peptide is clearly present for all ligands. 

**Figure 5:**
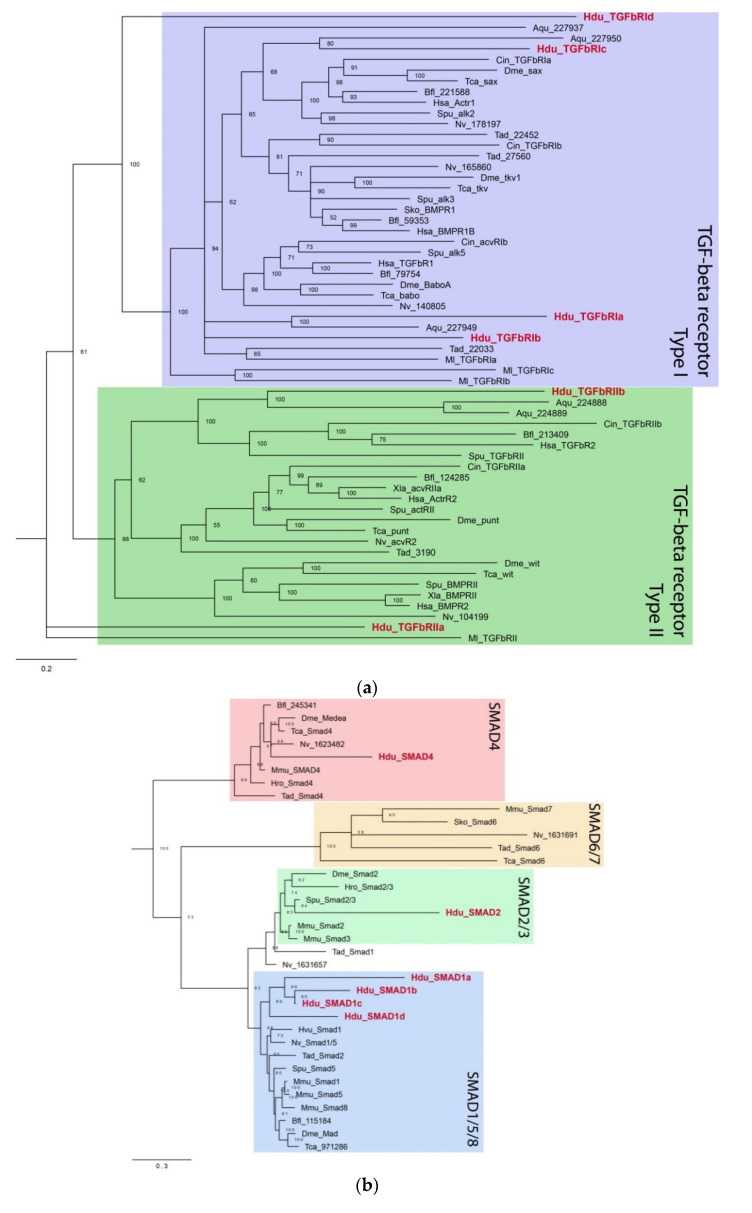
TGF-beta receptors (**a**) and SMADs (**b**). Unrooted Bayesian trees. *Halisarca dujardinii* sequences are highlighted in red. Numbers at three nodes indicate posterior probability.

There are two Type II receptors (*HduTGFbRIIa* and *HduTGFbRIIb*) and four Type I receptors (*HduTGFbRIa*–*HduTGFbRId*). All contain the extracellular receptor domain, single-pass transmembrane domain, and intracellular kinase domain. Additionally, all *H. dujardinii* Type I receptors possess the glycine–serine repeat (GS region) adjacent to the kinase domain, an arrangement that is characteristic of metazoan Type I receptors. Phylogenetic analyses included sequences of TGF-beta receptors from representative metazoans and showed that *H. dujardinii* receptors are not supported in individual receptor subclasses (Figure 5a). Instead, *HduTGFbRIIa* appears sister to all other Type II receptors and *HduTGFbRId* as a sister to all Type I subclasses. *HduTGFbRIc* together with *A. queenslandica* sequence form a weakly supported group with activating receptors Type I (sax/Actr1/alk2). *HduTGFbRIa* and *HduTGFbRIb* occupy the base of the Type I receptors subtree together with *A. queenslandica* and *M. leidyi* sequences. *HduTGFbRIIb* forms a well-supported clade with two *A. queenslandica* sequences, lancelet, human, sea urchin, and sea squirt Type II receptors.

We were also able to detect six SMAD proteins (Figure 5b). There are five receptor SMADs, one belonging to the SMAD2/3 family (*HduSMAD2*), and four SMADs forming a sister group to the SMAD1/5/8 clade (*HduSMAD1a*–*HduSMAD1d*). There is also a single Co-Smad (*HduSMAD4*). No inhibitory SMAD belonging to SMAD6 was observed. All SMADs have the predicted MH1 and MH2 domains, characteristic of SMAD proteins. 

### 3.3. Stages of Cell Reaggregation in Halisarca dujardinii

Cell reaggregation in *H. dujardinii* represents a development process leading from a suspension of single cells to a fully functional sponge. This process occurs through several highly stereotypic stages. However, cell reaggregation shows high plasticity in some aspects, leading to the heterogeneity of the aggregates. Firstly, the rate of cell reaggregation usually demonstrates variations, especially at later stages of the process. The rate of cell reaggregation varies not only in cell cultures obtained from different individuals but also within a single culture. That means that a culture will always represent an intermix of aggregates at slightly different developmental stages at a given time point. Another source of the aggregate heterogeneity is their size, as a culture usually includes aggregates varying from hundreds of micrometers to several millimeters in diameter.

**Figure 6:**
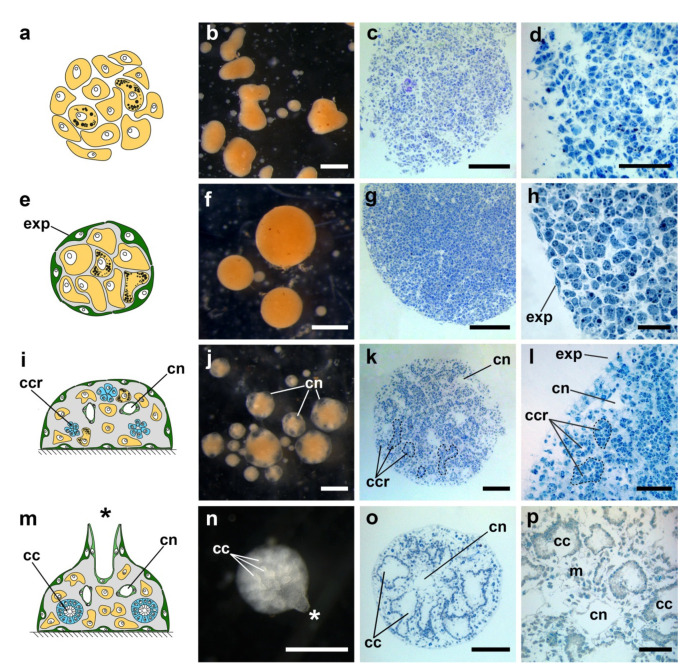
Key stages of the cell reaggregation and primmorph development in *Halisarca dujardinii*. (**a**–**d**) Primary multicellular aggregates; (**e**–**h**) True primmorphs, exp—exopinacoderm; (**i**–**l**) Progressed developing primmorphs, ccr—choanocyte chamber rudiments (marked by black dashed lines), cn—canals of aquiferous system, exp—exopinacoderm; (**m**–**p**) Reconstructed functional sponge, cc—choanocyte chambers, cn—canals of aquiferous system, m—mesohyl, osculum is marked by an asterisk; (**a**,**e**,**i**,**m**) Schematic representation of the developmental stages; (**b**,**f**,**j**,**n**) Developmental stages in vivo, stereomicroscopy, reflected light; (**c**,**g**,**k**,**o**) General histological structure, light microscopy, 1 µm thick sections; (**d**,**h**,**l**,**p**) Details of histological structure, light microscopy, 1 µm thick sections. Scale bars: (**b**,**e**,**h**,**k**) 500 µm; (**c**,**f**,**i**,**l**) 100 µm; (**d**,**l**,**p**) 50 µm; (**h**) 20 µm.

Nevertheless, the accurate in vivo studies supplemented with histological and ultrastructural investigation allow us to reliably identify several key and stereotypic stages of the cell reaggregation in *H. dujardinii* (Figure 6):

(1) Primary multicellular aggregates (PMAs), 0–24 h post-dissociation (hpd). The first small PMAs appear in a culture after 20–30 min of cultivation due to random contacts between single cells. During subsequent 24 h, they grow incorporating new cells or merging with each other. The distinguishable features of PMAs are an irregular shape, rough surface, and absence of any signs of internal structure, as PMAs represent a simple intermix of different cells.

(2) Early-stage primmorphs (ESPs) and true primmorphs (TPs), 1–3 days post-dissociation (dpd). Starting from 1 dpd, PMAs begin their transformation into ESPs and TPs. During the transformation, the aggregate surface is gradually covered by exopinacoderm, resulting in the isolation of the aggregate’s internal milieu. ESPs are aggregates with surface partially covered by exopinacoderm, while TPs are characterized by the complete exopinacoderm. As the rate of the exopinacoderm formation varies between aggregates, a culture after 1 dpd represents an intermix of aggregates at various stages of epithelization, including both ESPs and TPs. After 3 dpd, the epithelization in the vast majority of aggregates is complete, and culture predominantly contains TPs.

(3) Developing primmorphs (DPs), 5 + dpd. After a lag phase of various lengths, TPs start their progressive development to reconstruct functional sponges. This process begins with the formation of numerous small cavities in the internal parts of a primmorph. These cavities are the first sign of aquiferous system formation. Further, they give rise to canals of the aquiferous system.

(4) Progressed developing primmorphs (PDPs), 7 + dpd. As progressive development of primmorphs proceeds, rudiments of choanocyte chambers appear inside PDPs. These rudiments are dense spherical groups of cells, which will differentiate into choanocytes later on. Simultaneously, with the formation of choanocyte chamber rudiments, cavities gradually grow in size and obtain an endopinacocyte lining characteristic for mature canals of the aquiferous system.

(5) Reconstructed functional sponges (FSs), 10 + dpd. At this stage, cell reaggregation ends, and each aggregate transforms into a functional sponge with a complete aquiferous system: numerous ostia in exopinacoderm, elaborate net of canals and choanocyte chambers, and one or several oscular tubes.

### 3.4. RNA-Seq Analysis of Wnt and TGF-Beta Pathway Gene Expression

We studied the expression of the identified genes at two time points during cell reaggregation of *H. dujardinii*, at stages of ESP/TP, 1 day post dissociation (dpd), and DP, 6 dpd, using RNA-seq technology. These time points were chosen as the key stages of the cell reaggregation: the isolation of their internal milieu through the formation of the exopinacoderm occurs at 1 dpd; the development of an aquiferous system and other anatomical structures, which are probably underlain by re-establishment of positional information and determination of new organism polarity, occurs at 6 dpd. The bulk of aggregates from three independent cultures was taken for stages 1 dpd and 7 dpd. After sequencing, transcript quantification, and principal component analysis, one sample from each stage was omitted as highly diverged, and only two samples for each stage were processed for differential expression analysis.

**Figure 7:**
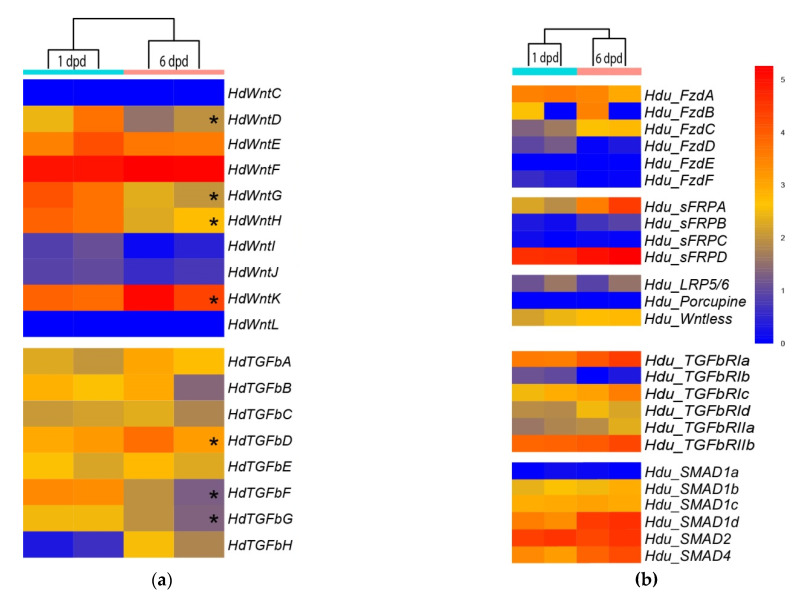
Heatmap representation of the expression profiles of the genes that participated in Wnt and TGF-beta signaling pathways during cell reaggregation in *Halisarca dujardinii*. Two time points in two replicates are shown. (**a**) *Wnt* and *TGF-beta* ligands expression. Transcripts with q < 0.05 between conditions are marked by asterisks (*). (**b**) *Frizzled*, *SFRP*, *LRP5/6*, *Porcupine*, *Wntless* expression in the upper part, and *TGFbRs* and *SMAD*s in the lower. The scale of the expression level is in transcript per million (TPM).

To visualize the expression of the identified *Wnt* and *TGF-beta* ligands, receptors, *LRP5/6*, *SMADs*, and some involved proteins during primmorph development, we generated a heatmap representation of expression. We were particularly interested in whether the elevation of *Wnt* and *TGF-beta* expression (including local) correlates with the interruption of radial symmetry during primmorph development. The majority of *Wnts* (8 out of 10) show no differences in expression between ESP/TP and DP stages (Figure 7a). Five genes (*HduWntD, HduWntE, HduWntI, HduWntJ, HduWntK*) are expressed at the medium level, two genes (*HduWntC*, *HduWntL*) at a low level, and one gene (*HduWntF*) at a high level at both stages (Figure 7a). In contrast, *HduWntG* and *HduWntH* show significant differences between studied stages, being represented in RNA of ESP/TP samples. 

*TGF-beta* does not show such dramatic differences for any transcript. Among them, two molecules are present at a higher level at the ESP/TP stage and another one at the DP stage.

Some differences in expression levels are also observed between paralogs of Frizzled, SFRP, TGF-beta receptors, and SMADs: *HduFzdA*, *HduSFRPD*, *HduTGFbRIa*, *HduTGFbRIIb*, and *HduSMAD2* are actively expressed at both analyzed stages, while *HduFzdD*, *HduFzdE*, *HduFzdF*, *HduSFRPC*, and *HduSMAD1a* are presented at minimal levels (Figure 7b). Intriguingly, the expression levels of *porcupine* and *Wntless*, proteins necessary for the Wnt secretion, differ by two orders of magnitude between each other but do not differ between the stages. *Dishevelled*, *TCF*, and *APC* are expressed at baseline levels, while *Groucho* and *GSK3b* are expressed at a high level. All of them show no differences between the developmental stages. Thus we demonstrate that some Wnt and TGF-beta molecules express during whole-body regeneration. Although their expression levels demonstrate wide variations even between replications, evidently expression level for the same signaling molecules change over time. Pay attention to the bulk nature of samples from primmorph culture, heterogeneity, and WMISH results (next paragraph) that we consider to carefully interpret quantitative data about *Wnt* and *TGF-beta* genes expression.

### 3.5. Wnt and TGF-Beta Expression during Primmorph Development in Halisarca dujardinii

To determine the role of Wnt and TGF-beta ligands in the development of *H. dujardinii* aggregates, we described the spatial patterns of their expression at different stages using WMISH. It was the first attempt to study spatial expression organization in primmorphs, and thus we focused on several key stages rather than checking all Wnt and TGF-beta genes at all stages of primmorph development. Stages 1, 3, 7, 10, and 24 dpd were investigated for *Wnt*, 5 and 9 dpd, for *TGF-beta* expression. Generally, *Wnt*, in contrast to *TGF-beta*, demonstrated a wide variety of expression patterns. However, we noted the heterogeneity of aggregates in the levels of Wnt and TGF-beta ligands’ expression. Cell aggregates of *H. dujardinii* show considerable size variations from the earliest stages of cell reaggregation [10]. We were able to show that large (700–800 µm) aggregates had diffuse or localized expression of most of the studied genes, while small ones (50–100 µm) most often did not show any signal. Therefore, further descriptions of expression patterns are given only for large aggregates.

**Figure 8:**
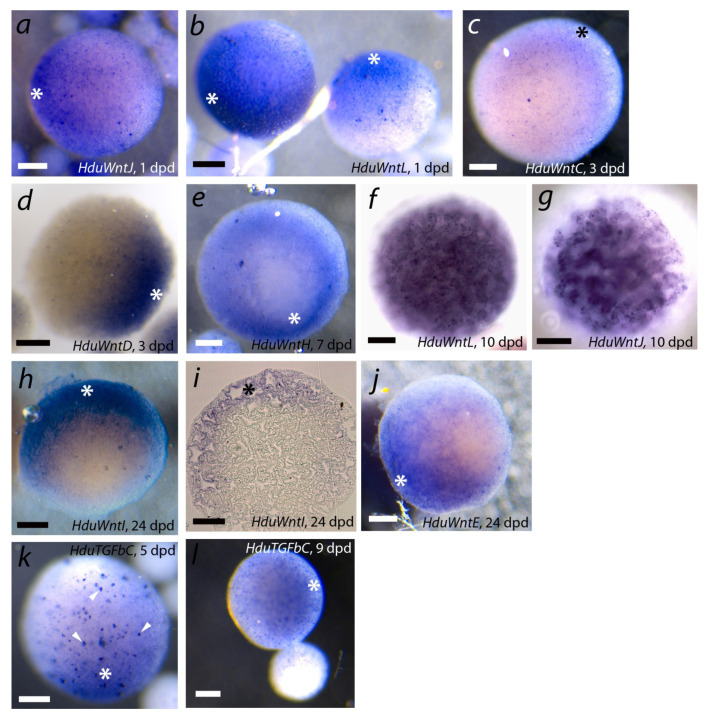
Expression of *Wnt* and *TGF-beta* at different stages of cell reaggregation in *Halisarca dujardinii*. Range of *Wnts* demonstrates asymmetric expression pattern from early stages of cell reaggregation to progressed stages of aquiferous system formation. (**a**–**d**) Early-stage primmorphs/true primmorphs; (**e**–**j**,**l**) progressed developing primmorphs; (**k**) developing primmorphs. (**a**–**e**,**h**,**j**–**l**) stereomicroscopy, reflected light; (**f**,**g**) stereomicroscopy, transmitted light; (**i**) light microscopy, 5 µm thick plastic section. Pole with highest expression level marked by asterisk. Large bright particles at (**k**) indicated by arrowheads are algae included in primmorph during reaggregation; this staining is non-specific. Scale bar is 150 µm.

Figure 8 represents some expression patterns demonstrating polarity in transcript localization. Diffuse patterns or absence of detectable expression are not shown for studied stages. We found asymmetric expression at different reaggregation stages for studied genes. Four genes (*HduWntC*, *HduWntD*, *HduWntJ,* and *HduWntL*) show asymmetric expression already during exopinacoderm formation at the ESP/TP stage (1–3 dpd) (Figure 8a–d). ESPs and TPs show the expression along the gradient with a maximum at one pole of the aggregate. These genes lose the polar expression at later stages: at the PDP stage (at 10 dpd), *HduWntD* is expressed diffusely, while *HduWntJ* and *HduWntL* are expressed in choanocytes of the emerging aquiferous system (Figure 8f,g).

For *HduWntH* and *HduWntI*, expression was studied only at later stages of cell reaggregation: in PDPs 7 dpd and 24 dpd. For both genes at both studied stages, an asymmetric expression with a maximum at one pole of the aggregate was shown (Figure 8e,h,i).

*HduWntE* has clearly asymmetric expression in the majority of studied PDPs at 24 dpd. This gene was expressed along most of the aggregate surface, except for one pole free of the transcript (Figure 8j). Intriguingly, at the earlier DP stage (6 dpd), the *HduWntE* transcript had uniformly high expression. Combined WMISH and RNA-seq data show that expression of *HduWntE* is gradually restricted during aggregate development: *HduWntE* show a high level of the expression in ESPs/TPs (1 dpd), then its expression level decreases in DPs (6 dpd), and, finally, *HduWntE* become vanished from one pole of DPDs (24 dpd).

Seven out of the eight studied *TGF-beta* demonstrated diffuse expression of varying intensity at all studied stages (1, 3, 6, 9, 12, and 20 dpd; Supplementary File S8). In contrast, *HduTGFbC* demonstrated a slight increase in expression at one pole of a primmorph at the DP (5 dpd) and PDP (9 dpd) stages.

Thus, we can state a dynamic pattern of asymmetric expression of individual *Wnt* and *TGF-beta* at different stages of *H. dujardinii* cell reaggregation.

## 4. Discussion

Both signaling mechanisms, Wnt and TGF-beta, are involved in the axis specification in a broad range of metazoan species. Most metazoans have at least one clearly defined axis in adulthood. The majority of sponges demonstrate a well-established morphological axis only at the stage of the swimming larva, and the involvement of the Wnt and TGF-beta ligands in embryonic development and larval patterning has been described only in *Sycon ciliatum* and *Amphimedon queenslandica* [7,8,9]. In contrast, the adult sponge has no axis in the usual sense—as the imaginary line joining two ends/sides of the body. However, at least one polar structure could be distinguished in any adult sponge—the osculum, exhalant aperture of the aquiferous system. It differs from other tissues both in structure and by molecular profile. It was shown that the osculum, due to the Wnt cascade activity, organizes the aquiferous system, i.e., there is a molecular mechanism that supports the animal’s body plan [13,37,38]. Moreover, cells constituting the osculum express *Wnt* and *TGF-beta* [9,13].

Sponges are capable of whole-body regeneration from dissociated cells into a functional adult animal in the course of cell reaggregation [11]. The molecular interactions that support the body plan should be lost during the tissue dissociation procedure and then be reestablished during subsequent aggregate development. This assumption is supported by observed morphological transformations of developing aggregates, which, in particular, change their symmetry: asymmetric primary multicellular aggregates develop into radially symmetric primmorphs and then into polarized reconstructed sponges, bearing osculum [10,39]. Differential expression of Wnt and TGF-beta was described during reaggregation of *S. ciliatum* cells by RNA-seq [12]. Considering these facts, we assumed that Wnt and TGF-beta pathways’ activity might take place in the polarization (appearance of asymmetry) of the developing primmorphs in *Halisarca dujardinii*.

The participants of Wnt and TGF-beta pathways in developmental processes have been previously described in another member of the class Demospongiae, *A. queenslandica*, and the calcareous sponge *S. ciliatum*. In the *Sycon*, 21 Wnt and 22 TGF-beta proteins were found, which dispelled the idea of only two homologs of Wnt ligand in the last common ancestor of multicellular organisms [8,9]. We isolated 10 homologs of Wnt from the *H. dujardinii* transcriptome and described dynamically expressed patterns in the adult sponge, larvae, during oogenesis and regeneration [13]. To reveal if the cascades are able to function, we annotate the main components of Wnt and TGF-beta pathways *H. dujardinii* transcriptome. Subsequent analysis of the involvement of Wnt and TGF-beta pathways in *H. dujardinii* primmorph development was done by the evaluation of gene expression using RNA-seq and describing ligand expression patterns in primmorphs by WMISH. Data about gene activity were compared with ultrastructural observations on the reaggregation and primmorph development in *H. dujardinii*.

The core components of the Wnt and TGF-beta signaling pathways in *H. dujardinii* demonstrate a high conservation level of protein sequence and domain architecture. The presence of critical amino acid residues in Frizzled and LRP5/6 suggests that most of the known protein–protein interactions that exist in the bilaterian Wnt pathway appear to be possible in *H. dujardinii*. Interestingly, Axin—the core protein of bCDC—was not identified in *H. dujardinii*. Axin was also not found in the *S. ciliatum*, although it is present in *A. queenslandica* [8,9]. Meanwhile, LRP5/6, which is responsible for binding to Axin in the cytoplasm, has all known conserved domains, as well as five cytoplasmic PPPSPxS motifs phosphorylated by GSK3b and CK1 (Figure 3a,c). Mutational analysis showed that substitution of serine to alanine in these motifs interferes with signal transduction into the cell, and LRP6, devoid of the extracellular domain, works as a constitutively active receptor. In addition, one motif out of five is sufficient for signal transduction [27,28,29,31,40,41]. It is the phosphorylated PPPSPxS motif that serves as the docking site for the cytoplasmic Axin [29]. In Bilateria, Axin binds GSK3b, CK1, beta-catenin, APC, and Dishevelled; it acts as a scaffold for efficient phosphorylation of beta-catenin. Perhaps, in sponges, Axin homologs do not play such a key role as in Bilateria, and some other protein performs its functions. However, in this case, the role of conservative sites of interaction with it on LRP5/6 remains unclear. Obviously, the structure of the Wnt pathway in sponges lacking Axin requires further study from a biochemical perspective. Thus, despite the absence of one of the main components of bCDC, we found sites for its binding in other proteins.

Meanwhile, despite the completeness of the description of the bCDC participants and their interactions at the molecular level, much in the functioning of this protein complex remains disputable. Moreover, it was shown that adhesion junction proteins are co-immunoprecipitated together with beta-catenin in *Ephydatia muelleri* but not participants of the Wnt pathway [42]. In contrast, in mammalian cells, bCDC components are co-immunoprecipitated together or with beta-catenin [43,44]. However, it seems that beta-catenin probably retains the conservative role as a substrate for GSK3b, since inhibition of GSK3b leads to the disappearance of phosphorylated beta-catenin [42]. 

Despite the presence of all conserved domains and critical amino acid residues, Frizzleds from sponges are closer to each other and orthologs from Ctenophora than to any of the described receptor families in Bilateria + Cnidaria. We can see that the Frizzleds’ sequences from sponges and comb jelly form a clade, sister to the Fzd/8 and Fzd1/2/7 branches. HduFzdA clusters into a subtree with two receptors from calcareous sponge *S. ciliatum* and ctenophore MlFzdB. HduFzdB is included in a compact group with sequences of other demosponges, *A. queenslandica*, *Suberites domuncula*, and *E. muelleri*. HduFzdC–HduFzdF, together with the proteins from *A. queenslandica* and *E. muelleri*, form a clade with ctenophoran MlFzdA. However, we must pay attention to the length of branches inside the clade of Porifera + Ctenophora sequences, considering the possible influence of long branch attraction. Based on this, we assume that Frizzled in sponges and ctenophores occurred independently of those of other multicellular organisms.

TGF-beta superfamily proteins together with Wnt participate in the specification of body axes and cell fates. These secreted pleiotropic factors play critical roles in embryogenesis and adult tissue homeostasis by regulating cell proliferation, differentiation, death, and migration in context-dependent manner. Mammal genomes contain 33 genes coding TGF-beta superfamily proteins from BMP, Activin, GDF, TGF-beta, and some other families. In *H. dujardinii*, we identified eight TGF-beta proteins, three of which could be classified as TGF-beta sensu stricto, six receptors, and six SMAD proteins. Type I and Type II receptors in *H. dujardinii* both demonstrate a high level of sequence divergence, although SMADs clearly fall into SMAD1/5/8, SMAD2/3, and SMAD4 families. Interestingly, representatives of SMAD6 or inhibitory SMAD were not found. SMAD proteins work in heteromeric complexes R-SMAD + Co-SMAD, and I-SMAD is a level of negative regulation of signaling activity. I-SMAD molecules are able to compete with Co-SMAD in binding with R-SMAD, not allowing the protein complex to activate the expression of target genes [33].

RNA-seq analysis of pathway components demonstrates differences in expression level for at least seven ligands: *HduWntD, HduWntG*, *HduWntH*, *HduWntK, HduTGFbD*, *HduTGFbF,* and *HduTGFbG*. Other components demonstrate different expression levels between paralogs as well. Basically, we observed similarities between replicas; however, for a few transcripts (*HduFzdB*, *HduLRP5/6*), striking differences between replicas of the same stage were observed. We suppose that this results from the heterogeneity of the primmorphs population, which usually shows a slightly different rate of development even within a single culture [10,45]. Nevertheless, 5 out of 18 ligands Wnt and TGF-beta demonstrate significant differences in expression level between the studied stages of cell reaggregation. Attention is drawn to the scatter of expression levels within the *Wnt* and *TGF-beta*: the map shows only relative values, making some transcripts seem close to the zero expression level. However, these transcripts are expressed during primmorph development, which is confirmed by the WMISH data.

Our first experiments of WMISH with probes to *Wnt* and *TGF-beta* demonstrate polar and gradient expression for several signaling molecules in primmorphs. Most of *TGF-beta* express uniformly at high or low levels at studied stages from early-stage primmorphs to progressed developing primmorphs (1, 3, 6, 9, 12, and 20 days post dissociation), but *HduTGFbC* demonstrates a gradient pattern of expression in progressed developing primmorphs. Uniform expression of several *Wnt* genes was not documented in Figure 7, although several *Wnt*s such as TGF-beta express uniformly at different stages and levels that are demonstrated by RNA-seq. We found broad gradient patterns for some *Wnt* and *TGF-beta* with stock at one primmorph pole already at 24 hpd. It differs from similar experiments with reaggregation of *Hydra* cells where expression of *Wnt* starts from a cluster of several cells [46].

## 5. Conclusions

Changes in the expression levels of the Wnt and TGF-beta ligands during the primmorph development, dynamically changing their expression patterns with a sign of polarity, described the participation of these signaling cascades in the axes specification in development allow us to assume that the formation of new axes occurs in primmorphs through the involvement of the Wnt and TGF-beta pathways. Whether they are the first elements in the chain of molecular patterning of a newly forming organism, what events link their activation with the formation of polarized body structures, and how the pattern arises from ubiquitous expression are questions that remain to be answered.

## Data Availability

Nucleotide sequences described in the article are in GenBank with accession numbers MZ042492-MZ042530.

## Supplementary Materials

The following are available online at https://www.mdpi.com/article/10.3390/genes12060944/s1, Supplementary File S1: Alignment of Frizzled proteins in FASTA format, Supplementary File S2: Alignment of LRP proteins in FASTA format, Supplementary File S3: Alignment of TGF-beta proteins in FASTA format, Supplementary File S4: Alignment of TGF-beta receptors in FASTA format, Supplementary File S5: Alignment of SMAD proteins in FASTA format, Supplementary File S6: List of sequences’ accession numbers used in analyses. File S7: Predicted 3D and domain structure of *HduLRP5/6*.Supplementary File S8: Additional samples for WMISH experiments.
