# Supplementary material for: Expression of Wnt and TGF-Beta Pathway Components during Whole-Body Regeneration from Cell Aggregates in Demosponge Halisarca dujardinii
Source: Genes (Basel). 2021 Jun 20;12(6):944. doi: 10.3390/genes12060944 (PMC8235796; doi:10.3390/genes12060944)
Supplement: Supplementary file 1 [file genes-12-00944-s001.zip › Supplementary File 6.pdf]

Supplementary File 6. List of accession numbers for sequences used in analysis.

**WNT**	
HduWntC	CUW00361.1
HduWntD	CUW00360.1
HduWntE	CUW00365.1
HduWntF	CUW00358.1
HduWntG	CUW00357.1
HduWntH	CUW00359.1
HduWntI	CUW00356.1
HduWntJ	CUW00364.1
HduWntK	CUW00362.1
HduWntL	CUW00363.1
**FRIZZLED**	
AquFzdA	ADO16569.1
AquFzdB	ADO16570.1
CheFzd1	ABI98898.1
CheFzd2	AFI99118.1
CheFzd3	ABI98899.1
CheFzd4	AFI99119.1
DmeFzd1	P18537
DmeFzd2	Q9VVX3
DmeFzd3	O77438
DmeFzd4	Q9NBW1
GdFzd10	Q9PWH2
GdFzd2	Q9IA06
GdFzd4	Q9IA05
GdFzd7	O57329
GdFzd8	Q9IA03
GdFzd9	Q9IA02
MlFzdA	ADO34161.1
MlFzdB	ADO34162.1
MmFzd1	O70421
MmFzd10	Q8BKG4
MmFzd2	Q9JIP6
MmFzd3	Q61086
MmFzd4	Q61088
MmFzd5	Q9EQD0
MmFzd6	Q61089
MmFzd7	Q61090
MmFzd8	Q61091
MmFzd9	Q9R216
NvFzd1	XP_001647540.1
NvFzd3	EDO38567.1
NvFzd4	XP_001622965.2
NvFzd5	XP_001634995.1
NvFzd9	XP_032235151.1
SciFzdA	CDO67909.1
SciFzdB	CDO67910.1
SciFzdC	CDO67911.1

SciFzdD	CDO67912.1
SdoFzd	Q70LH3
XlFzd1	Q9I9M5
XlFzd10A	Q9DEB5
XlFzd10B	Q9W742
XlFzd2	Q9PUU6
XlFzd3	O42579
XlFzd4	Q9PT62
XlFzd5	P58421
XlFzd7A	Q9PUK8
XlFzd7B	Q8AVJ9
XlFzd8	O93274
**LRP**	
DmeArrow	Q95V09
LRP_CAEEL	Q04833
LRP1_HUMAN	Q07954
LRP1_MOUSE	Q91ZX7
LRP1_RAT	G3V928
LRP10_HUMAN	Q7Z4F1
LRP10_MOUSE	Q7TQH7
LRP11_MOUSE	Q8CB67
LRP12_HUMAN	Q9Y561
LRP12_MOUSE	Q8BUJ9
LRP12_PONAB	Q5R662
LRP2_HUMAN	P98164
LRP2_MOUSE	A2ARV4
LRP2_PIG	C0HL13
LRP2_RAT	P98158
LRP3_HUMAN	O75074
LRP3_RAT	O88204
LRP4_HUMAN	O75096
LRP4_MOUSE	Q8VI56
LRP4_RAT	Q9QYP1
LRP5_HUMAN	O75197
LRP5_MOUSE	Q91VN0
LRP5L_HUMAN	A4QPB2
LRP6_HUMAN	O75581
LRP6_MOUSE	O88572
LRP8_CHICK	Q98931
LRP8_HUMAN	Q14114
LRP8_MOUSE	Q924X6
NvLRP5/6	EDO49133.1
**TGF-beta**	
Aca_nodl	ACM50754.1
Ame_act	XP_016907093.2
Ame_admp	XP_016767338.2
Ami_dpp	XP_029191188.1
Bfl_80287	XP_035670031.1
Bfl_Bmp24	AAC97488.1
Bfl_Bmp3	XP_019639535.1

Bfl_Mstn	XP_035688130.1
Bfl_nodr	AAL99367.1
Bfl_Tgf	XP_035697090.1
Bfl_Vg1	ACF94997.1
Bfl244225	XP_035699471.1
Bfl99205	XP_035678629.1
Bgl_nod	XP_013067923.1
Bmi_dpp	NP_001138801.1
Cel_cet1	AAC26791.1
Cgi_gdf3	CAD67715.1
Cin_admp	NP_001071985.1
Cin_Tgfb2	NP_001071838.1
Cs_lefty	BAB68348.1
Dme_act	AAL51005.1
Dre_actBb	CAB43092.1
Dre_admp	NP_571951.2
Dre_Bmp10	NP_001124072.1
Dre_Bmp6	XP_021325833.1
Dre_Dvr1	XP_017207084.1
Dre_gdf7	AAD20829.1
Dre_gdnf	Q98TU0.1
Dre_inhBb	NP_001018166.1
Dre_lefty	AAD34388.1
Dre_lefty2	AJG05927.1
Dre_mstn	AAB86693.1
Dre_mstn2	AAI63304.1
Dre_Tgfb1	NP_878293.1
Dre_Tgfb2	NP_919366.1
Dre_Tgfb3	NP_919367.2
Hm_Bmp58	AID65996.1
Hro_nod	BAC11909.1
Lgi_nodal	ACB42423.1
Mm_actBc	BAE20892.1
Mm_bmp10	AAI45045.1
Mm_bmp3	NP_775580.1
Mm_Bmp6	XP_035311489.1
Mm_gdf10	AAH22669.1
Mm_gdf2	4MPL_A
Mm_gdf3	XP_006505603.1
Mm_gdnf	NP_001288262.1
Mm_InhBe	NP_032408.2
Mm_neurt	NP_032764.1
Mmu_artemin	NP_445849.1
Mmu_persephin	O70300.1
Nv_act	ABF61781.1
Nv_Bmp24	AAR13362.1
Nv_Bmp58	ABC88372.1
Nv_gdf5	AAR27581.1
Nv_mst	XP_001641598.2
Nve_1639178	EDO47115.1

Pca_Bmp58	ABA42602.1
Pdu_dpp	CAJ38807.1
Sja_TGFb1	ADH10175.1
Sko_activin	NP_001161496.1
Sko_admp	NP_001158394.1
Sko_Bmp24	NP_001158387.1
Sko_bmp3	XP_002735398.1
Sko_Bmp58	NP_001158388.1
Sko_lefty	ALR88590.1
Sko_nodalA	ACY92597.1
Sko_nodalB	NP_001161612.1
Sko_nodalC	NP_001164721.1
Sko_TGFb2	ADB22639.1
Sko_univinlike	ACY92678.1
Spu_actB	NP_001121540.1
Spu_Bmp24	ACA04460.1
Spu_bmp3	XP_030834962.1
Spu_Bmp58	NP_999820.1
Spu_gdf11	XP_030846390.1
Spu_lefty	NP_001123281.1
Spu_nod	NP_001091919.1
Spu_uni	XP_030854149.1
Tad_57057	XP_002113173.1
Tad_57877	XP_002113941.1
Tad_58663	XP_002114631.1
Tad_9129	XP_002114186.1
Tad_9164	XP_002114398.1
Tca_actl	XP_970355.1
Tca_bmp10	XP_973577.1
Tca_myo	XP_966819.1
Tsp_Tgh3	OUC40670.1
Xl_actD	NP_001079333.1
Xl_Admp1	NP_001081792.1
Xl_admp2	NP_001090587.1
Xl_Bmp2	NP_001095136.1
Xl_Bmp3	NP_001082633.1
Xl_der	NP_001080966.1
Xl_Gdf5	NP_001086466.1
Xl_Gdnf	NP_001090196.1
Xl_lefty	NP_001082043.1
Xl_Tgfb2	CAA36117.1
Xl_Vg1	AAW30007.1
Xl_Xnr2	NP_001081436.1
Xl_Xnr6	NP_001079033.1
**TGF-beta receptors**	
Aqu_224888	XP_003384911.3
Aqu_224889	XP_003384911.3
Aqu_227937	XP_019860622.1
Aqu_227949	XP_011409575.2
Aqu_227950	XP_003383433.2

Bfl_124285	XP_035657497.1
Bfl_213409	XP_035657408.1
Bfl_221588	XP_035661292.1
Bfl_79754	XP_035661061.1
Cin_acvRIb	NP_001093904.1
Cin_tgfbRIa	NP_001071834.1
Cin_tgfbRIb	NP_001071968.1
Cin_TGFbRIIa	NP_001071835.1
Cin_TGFbRIIb	NP_001071836.1
Dme_BaboA	NP_477000.1
Dme_punt	NP_001262575.1
Dme_sax	AAA53242.1
Dme_tkv1	XP_033172871.1
Dme_wit	AAL16073.1
Hsa_AcvR1	XP_028364717.1
Hsa_AcvR2	XP_016863005.1
Hsa_BMPR1B	NP_001243722.1
Hsa_BMPR2	XP_011509989.1
Hsa_TGFbR1	NP_004603.1
Hsa_TGFbR2	JC5373
Ml_TGFbRIa	AEP16397.1
Ml_TGFbRIb	AEP16398.1
Ml_TGFbRIc	AEP16399.1
Ml_TGFbRIIR	AEP16396.1
Nv_104199	XP_032238543.1
Nv_140805	XP_032225445.1
Nv_165860	XP_001633896.1
Nv_178197	AFP87425.1
Nv_AcvR2	AAS77521.1
Sko_BMPR1	NP_001158366.1
Spu_AcvRII	XP_030828527.1
Spu_alk2	XP_030852853.1
Spu_alk3	XP_797469.4
Spu_alk5	XP_798456.4
Spu_BMPRII	XP_790983.2
Spu_TGFbRII	XP_030828751.1
Tad_22033	XP_002110855.1
Tad_22452	XP_002110322.1
Tad_27560	XP_002114481.1
Tad_3190	XP_002116607.1
Tca_babo	KYB28937.1
Tca_punt	XP_008198742.1
Tca_sax	NP_001164080.1
Tca_tkv	EFA09250.1
Tca_wit	XP_974821.1
Xla_AcvRIIa	NP_001084061.1
Xla_BMPRII	AAI70174.1
**SMADs**	
Bfl_115184	XP_035681878.1
Bfl_245341	XP_035671187.1

Dme_Mad	P42003.1
Dme_Medea	AAC35436.1
Dme_Smad2	NP_001285006.1
Hro_Smad2_3	BAB87720.1
Hro_Smad4	BAB87723.1
Hvu_Smad1	NP_001296671.1
Mbre_MH2	XP_001743193.1
Mmu_SMAD4	P97471.2
Mmu_Smad1	P70340.2
Mmu_Smad2	Q62432.2
Mmu_Smad5	P97454.2
Mmu_Smad7	O35253.1
Nv_1623482	EDO31382.1
Nv_1631691	EDO39628.1
Sko_Smad6	NP_001158449.1
Tad_Smad1	XP_002108907.1
Tad_Smad2	XP_002112850.1
Tad_Smad4	XP_002116214.1
Tad_Smad6	XP_002108167.1
Tca_971286	XP_971286.2
Tca_Smad4	XP_971429.2
Tca_Smad6	NP_001139378.1
