# Supplementary material for: Expression of Wnt and TGF-Beta Pathway Components during Whole-Body Regeneration from Cell Aggregates in Demosponge Halisarca dujardinii
Source: Genes (Basel). 2021 Jun 20;12(6):944. doi: 10.3390/genes12060944 (PMC8235796; doi:10.3390/genes12060944)
Supplement: Supplementary file 1 [file genes-12-00944-s001.zip › Supplementary File 7.pdf]

Supplementary File 7. (a) 3D model of 1st beta-propeller domain of HduLRP5/6. Yellow arrows correspond to beta sheets, red helix – alpha helix. Six blades of beta propeller, each of four beta sheets, are recognizable. Tertiary structure predicted with RaptorX (http://raptorx.uchicago.edu/) according: Xu, J. Distance-based protein folding powered by deep learning. PNAS August 20, 2019, vol. 116 (34), p. 16856-16865. doi.org/10.1073/pnas.1821309116

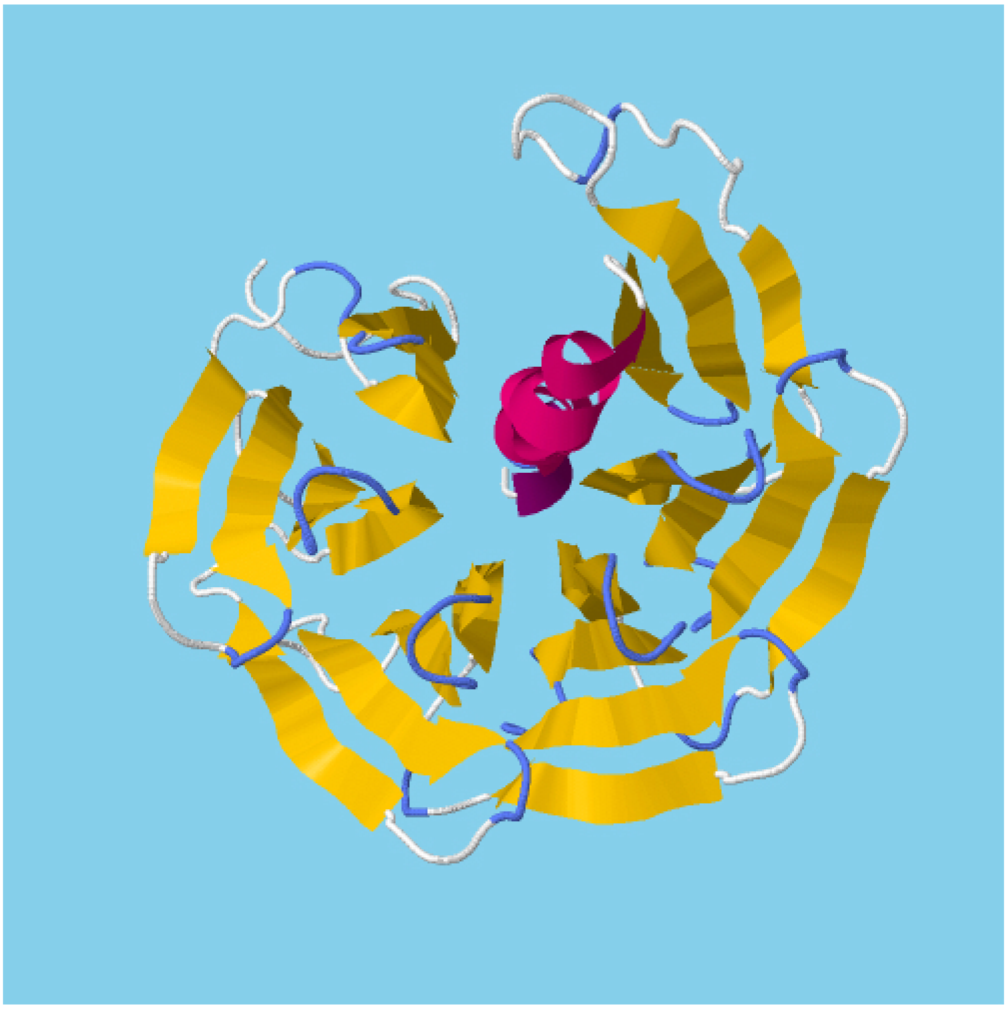

Supplementary File 7. (b) Secondary structure of HduLRP5/6 predicted with JPred4 [26]. Green arrows (jnetpred) indicate beta sheets according consensus prediction, based on alignment and hidden Markov model searching prediction; red coils - helixes. Blue ribbons indicate domains recovered by HMMSCAN against Pfam database. The diagram below (JNETCONF) demonstrate the confidence estimate for the prediction

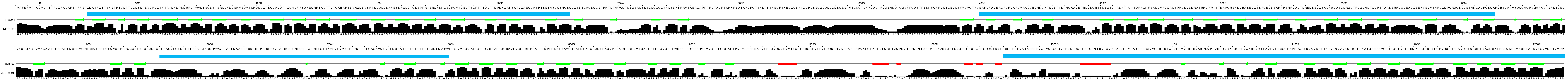
jnetpred
JNETCONF
jnetpred
JNETCONF
