# Supplementary material for: Expression of Wnt and TGF-Beta Pathway Components during Whole-Body Regeneration from Cell Aggregates in Demosponge Halisarca dujardinii
Source: Genes (Basel). 2021 Jun 20;12(6):944. doi: 10.3390/genes12060944 (PMC8235796; doi:10.3390/genes12060944)
Supplement: Supplementary file 1 [file genes-12-00944-s001.zip › Supplementary File 8.pdf]

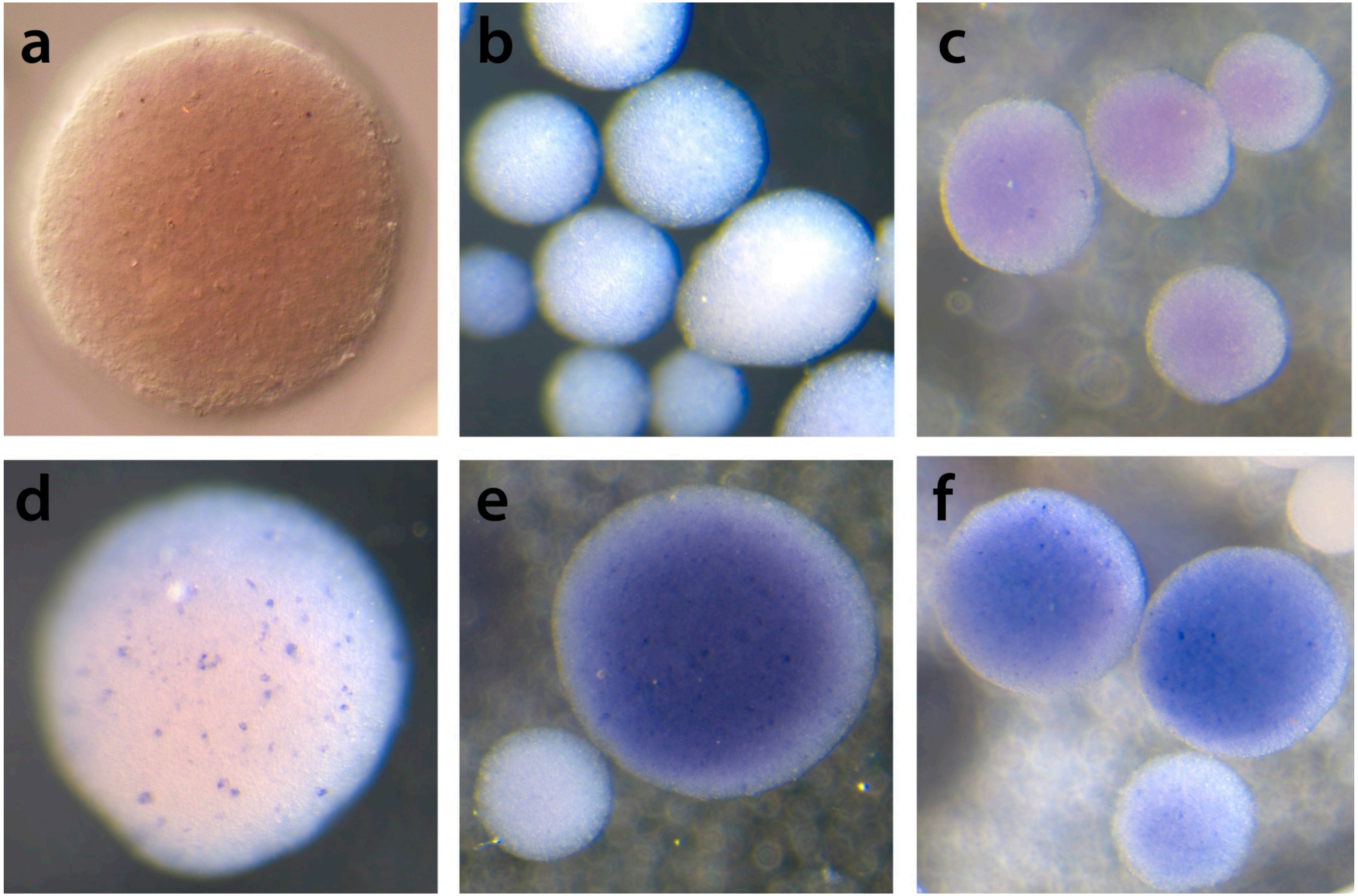

Supplementary File 8. (a-d) negative controls, (e-f) diffuse patterns of expression. Samples were hybridized with sense *HduWntL* (a, b) or sense *HduWntD* (c, e) probe as described in Material and Methods section. Color developed at room temperature overnight (a, b, d) or at 37°C during 24 h. Note artificially stained dark dots at (d) – the algae. (e) antisense probe to *HduWntK*, 1 dpd, and (f) antisense probe to *HduTGFbG*, 3 dpd, demonstrate diffuse staining pattern. Staining development during 5 h at room temperature. Note the little primmorph clear of staining. (a) DIC, (b – f) stereomicroscopy, reflected light.
